# A Stone in the Kidney

**Published:** 1889-05-04

**Authors:** 


### Within the Wards.

### A STONE IN THE KIDNEY.

A stone or several stones in one or both kidneys are not very uncommon, and are generally both painful and dangerous. Not very long ago the attempt to cure this condition by operation was considered unjustifiable, and the case was treated by palliatives only. It is very seldom that a patient can be made to see himself exactly as the doctor sees him. The physician regards him dispassionately and scientifically, and gives the justest verdict he can after a careful review of the evidence. If that evidence be unfavourable to the patient, he not unnaturally hopes that the judgment of the physician or surgeon may be wrong. He therefore resolves to try another, and another, and yet another medical man, or non-medical pretender, in the hope that one or other of them may be able to relieve his pain and to save his life. How very natural! From the doctor's point of view it is, no doubt, often a foolish waste of time and money. But what limits shall be set to the expedients of despair?

The case to be related is full of instruction to all classes of readers. X. was 58 years of age, and a man of education and position. He had been seriously ill for 30 years. His habits had been of a very sedentary character, both before his illness commenced and afterwards. He was a confirmed dyspeptic. His dyspepsia gave rise to painful and distressing flatulence, and was said by the doctors to be mainly due to rich living and want of exercise. How often he had been warned against both the rich living and the sedentary habits during the 30 years of his illness, who shall say? He had constantly a foul and bitter taste in his mouth, habitual constipation, and such nausea that the sight or even the smell of food would make him sick; and he was a martyr to thirst. By day the pain was severe and almost constant; by night the rest was broken both by pain and the imperative necessity of frequently rising. Life could not have been other than miserable, and yet the patient seems to have obstinately adhered during the whole time both to his rich living and his sedentary habits. It is more than probable that he was one of those men who, because they had not cured him, despised the doctors and all their works.

X. at last heard of the operation of "nephrolithotomy," or the removal of stone from the kidney. He appears to have decided in his own mind that this was the very operation he needed, and accordingly he made application in the proper quarter in order that it might be done without delay. The surgeon who was to perform the operation advised him to wait for a month, to leave off his sedentary habits, and to live and diet himself by a fixed rule. A surgical colleague counselled waiting and regular living for three months rather than one. At the end of three weeks a complete holiday and regular living had to a large extent cured the gouty dyspepsia; but the weary pain in the loin was unabated, and walking and driving exercise were both difficult and distressing. Instead of remaining at his health resort for three months as advised, he returned to London at the end of one, determined to have the operation performed forthwith. Yielding, therefore, to his resolute urgency, the surgeon consented to the operation and fixed the day.

It is not necessary to follow the operator's proceedings in detail. The diagnosis proved to be quite correct, for on making the incision a large hard mass was felt in the position of the pelvis, and in the very middle of the kidney. The stone, on being exposed, was loosened in its bed, and then extracted by means of the lithotomy forceps. It weighed no less than 473 grains, and is, therefore, one of the largest which has ever been removed from the human subject. Strange to say, except that it was thinned in parts, the kidney seemed practically unaltered. The patient rallied well from the operation, but for the first three days the normal use of the kidneys seemed to be a good deal embarrassed. The appetite was fairly good, and the spirits were cheerful. But his old enemy flatulent dyspepsia worried him both by day and night, and gave him very little peace.

If ever one man should render to another an implicit and unquestioning obedience, it is during the few days or weeks immediately succeeding a dangerous surgical operation. When a patient has made up his mind to submit to such an operation, he should select a surgeon in whom he has the fullest confidence; and having selected him, he should give himself up to medical direction with the completest effacement of his own will. This is an imperative condition if the best results are to be obtained. The failure to fulfil the condition may cost the patient his life. X. appears to have been a man obstinate by nature. His thirty years' suffering had probably taught him many little methods of relief, and made him, to some extent, have faith in himself as "his own physician." At any rate, instead of submitting entirely to medical guidance, he was obstinately bent upon having a daily action of the bowels. He insisted upon having the bedpan, and on one day attempted to use it no less than thirty times in the twenty-four hours, although there was no necessity for it. On any day that the bowels did not act to his satisfaction, he refused solid food of every kind, and took nothing at all but barley-water. There was thus no gain of strength, although until the seventh day he held his own fairly well.

On the seventh day there was a marked change for the worse. The patient got very restless, and the flatulence was distressing and uncontrollable. The eighth day brought no relief of symptoms. The restlessness could not be abated, and the strength began markedly to fail. Then the mind wandered, and was possessed by constant hallucinations. Finally, the dreaded symptoms of coma began to appear, with twitchings of the muscles and of the limbs. Death was now imminent, and on the morning of the ninth day, with gradually deepening coma, the patient ceased to breathe. This case is not recorded as a triumph of modern surgery, but as illustrating some of the many difficulties of medical and surgical practice, and as showing the extreme folly and danger of want of docility in a critical emergency. It is not affirmed that the patient would have made a good recovery if he had yielded himself entirely to the directions of the doctors, though many patients do recover from the same kind of operation as he underwent. But it is said that his persistent determination to have his own way interfered with the treatment to such an extent as to render it tenfold more difficult and dangerous. No infallibility is or ought to be claimed for medical men under any circumstances, but common-sense and experience alike show that they know their work so well, and so infinitely better than the patient, that the best hope of recovery undoubtedly lies in the most implicit obedience. Had X. yielded to them instead of taking his own way, it is possible he might now have been alive and comparatively well.

